# Appendiceal Adenocarcinoma Presenting As Perforated Appendicitis

**DOI:** 10.7759/cureus.13578

**Published:** 2021-02-26

**Authors:** Sam Stein, Benjamin Raymond D.O.

**Affiliations:** 1 General Surgery, United Hospital Center, Bridgeport, USA; 2 General Surgery, West Virginia School of Osteopathic Medicine, Lewisburg, USA

**Keywords:** intra-abdominal adhesions, appendiceal adenocarcinoma, colorectal cancer, cancer metastasis, perforated appendicitis, peritoneal pseudomyxoma, appendicitis, ileostomy

## Abstract

Primary tumors of the appendix, specifically appendicular adenocarcinoma, are a rare malignant neoplasm of the gastrointestinal tract. We present a case of a 64-year-old female who had significant peritoneal adhesions from a previously perforated appendix involving the right ureter, bladder, and anterior abdominal wall after a course of perforated appendicitis, which was managed conservatively with drain placement with interval appendectomy. We are discussing this case in hopes of bringing awareness to the possibility of underlying malignancy in the setting of perforated appendicitis in patients of advanced age.

## Introduction

Appendiceal cancers are extremely rare and difficult to diagnose prior to surgical intervention. According to the National Cancer Institute, these cancers make up 0.4% of all gastrointestinal malignancies [1]. The average age at diagnosis is 50 years. They also occur in a large variety of subtypes with neuroendocrine or carcinoid cancers being the most common. Neuroendocrine cells arise from specialized enterochromaffin cells that reside within the wall of the intestine and facilitate gastrointestinal motility and digestion [2]. Epithelial cancers of the appendix are less frequent and arise from gland-forming cells that line the inside of the appendix. Carcinoids are endocrine cell tumors that fall within the epithelial subclass and often present within recurrent right lower quadrant pain that can mimic acute appendicitis [1]. These cancers are then further subcategorized based on the cells detected microscopically and whether there is an invasion of the appendiceal wall. These include the goblet cell carcinoid, low-grade mucinous neoplasm, high-grade mucinous neoplasm, and adenocarcinomas. The subcategory of focus in this case, adenocarcinoma, is further classified as mucinous, signet-ring cell, and undifferentiated. These subclasses are essential for determining prognosis and treatment. After starting in the appendix, these malignancies can often spread outside the wall of the appendix and ultimately into the abdominal cavity. This can, at times, lead to a build-up of mucinous fluid in the abdomen known as pseudomyxoma peritonei or growth into the abdominal cavity known as peritoneal carcinomatosis. Treatment varies depending on the stage of the disease and its specific subtype [2]. Treatment may be complicated by peritoneal adhesions especially in the setting of previous abdominal surgery, trauma, or prior perforation. Focusing on increasing the patient's survival and understanding the capabilities and limitations of the hospital in which the operation is taking place is crucial when anatomical anomalies or complications arise intra-operatively.

## Case presentation

We report a case of a healthy 64-year-old female who originally presented to an outside emergency department with low-grade fever and a two-week history of worsening lower abdominal pain. She denied any nausea, vomiting, loss of appetite, diarrhea, constipation, melena, hematochezia, urinary dysuria, frequency, vaginal bleeding, or discharge. She denied any family history of colon cancer or inflammatory bowel disease. Past surgical history includes tubal ligation, and past medical history includes hypercholesterolemia, hypothyroidism, and lung adenocarcinoma years ago that resulted in a left lobectomy. She reported having a previous colonoscopy many years ago without any signs of polyps or masses.

**Figure 1:**
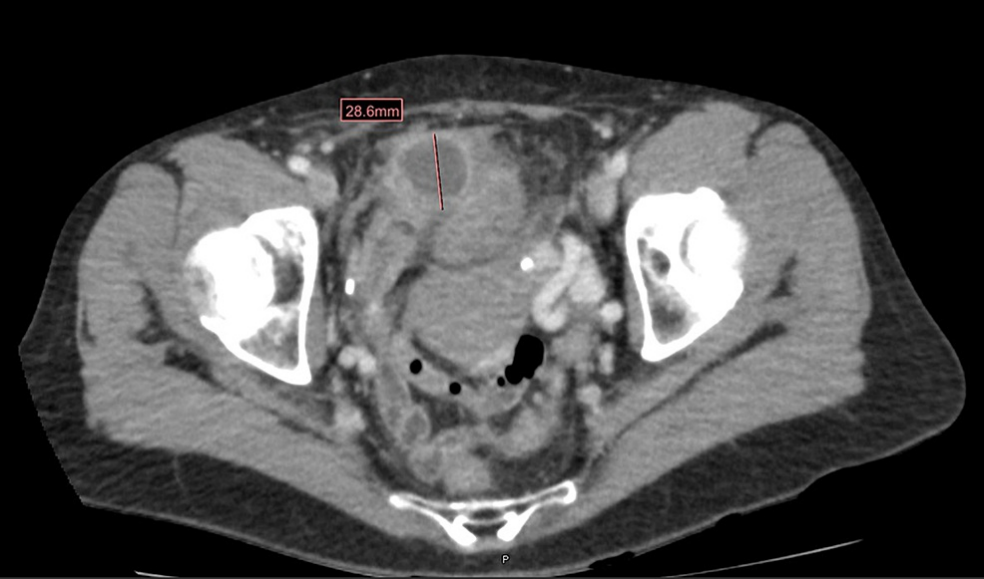
Perforated appendicitis with an abscess in the right lower quadrant. Red line shows abscess measuring 28.6 mm.

Vitals included height 1.676 m (5' 6"), weight 57 kg (126 lbs.), and BMI 20 kg/m². Physical exam was benign except for the following findings: fever of 38.0°C, moderate-to-severe tenderness with rebound and guarding in the lower abdomen and suprapubic region (right greater than left), and positive McBurney's point. As seen in Figure 1, the CT scan obtained in the emergency department showed findings concerning perforated appendicitis with subsequent abscess formation in the right lower quadrant.

**Figure 2:**
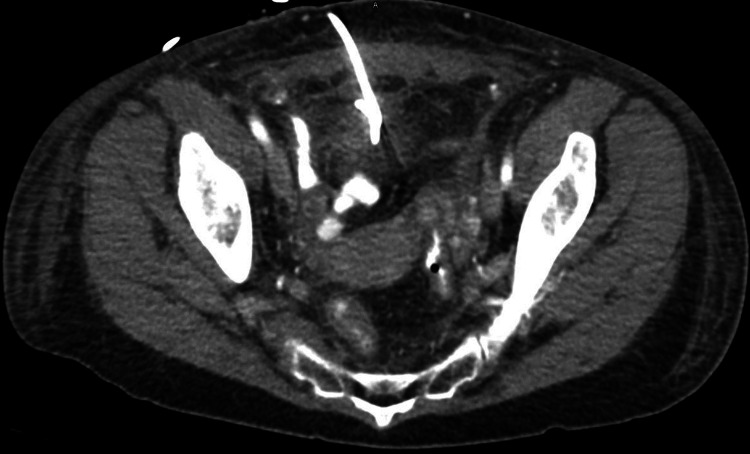
Perforated appendicitis with percutaneous drain in place.

Once the diagnosis was confirmed clinically and via radiographic imaging, she was transferred for definitive surgical and medical management. On hospital day 1, a percutaneous drain was placed with radiographic assistance with the return of purulent and serosanguineous fluid. Over the following days, her diet was slowly advanced and a repeat CT scan was obtained a few days after the original, as seen in Figure 2, which showed resolution of the right lower quadrant abscess collection. The drain was removed at that time, and the patient was discharged home with a course of oral antibiotics and plans for colonoscopy evaluation and ultimately an interval appendectomy.

The patient was seen three weeks post inpatient hospitalization to discuss the possible surgical intervention. At that time, the patient was feeling much better and tolerating a normal diet. She had appropriate bowel and bladder function and denied any pain, redness, fever, chills, or gastrointestinal symptoms. On physical exam, the abdomen was soft and supple without any tenderness, distention, or masses present. The patient agreed on obtaining a colonoscopy in four weeks, which aligned with the standard of care of obtaining one 6-8 weeks after an acute perforation of the appendix.

**Figure 3:**
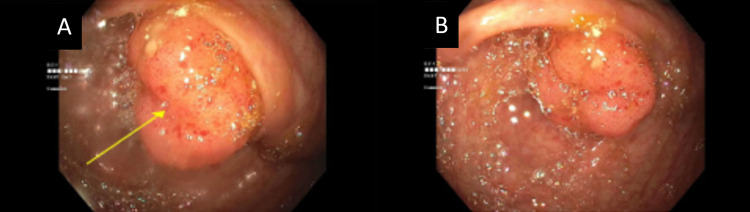
Colonoscopy reveals a fungating, non-obstructing, medium-sized mass at the appendiceal orifice. (A) Yellow arrow marks mass present at the appendiceal orifice. (B) Additional image from colonoscopy further demonstrating mass.

**Figure 4:**
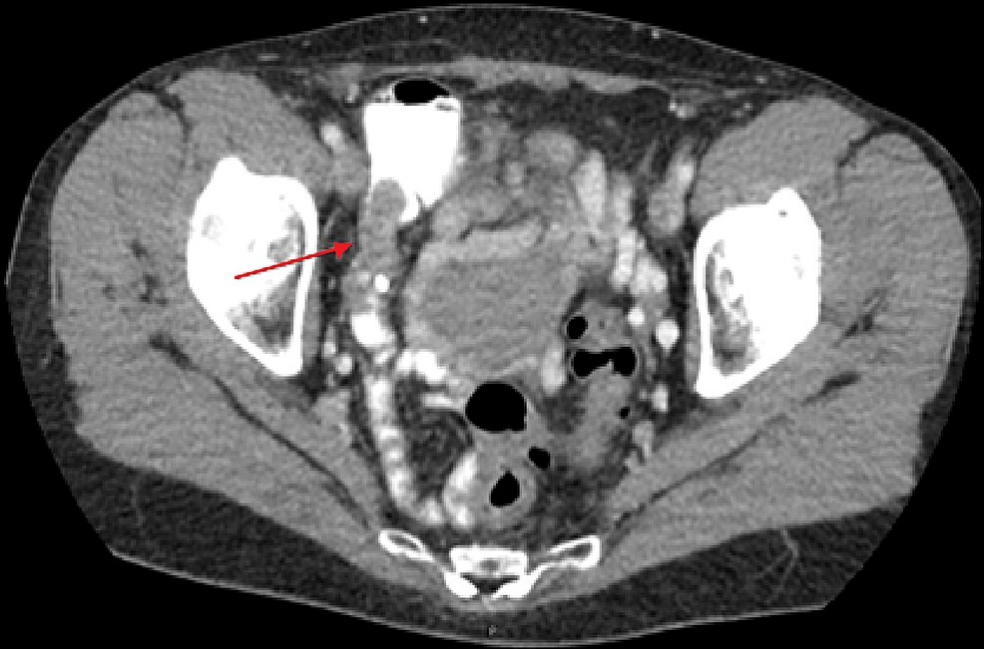
Staging CT scan prior to surgical intervention shows no signs of metastatic disease. Red arrow shows mass in the appendix extending into the colon.

Figures 3A, 3B demonstrate the colonoscopy findings that showed a tumor at the appendiceal orifice with pathology consistent with that of a tubulovillous adenoma. The patient met the surgeon the following week to discuss the findings and agreed on a right laparoscopic hemicolectomy in four weeks for a cancerous peri-appendiceal mass. Figure 4 illustrates a staging CT scan that was obtained prior to the operation.

Laparoscopic exploration of the abdominal cavity revealed evidence of a grossly inflamed appendix with the adhesion of the appendiceal tip to the anterior abdominal wall, bladder dome, and right ureter. The surgeon performed a partial appendectomy with associated right hemicolectomy for oncological purposes with clip placement at the anterior abdominal wall for future identification of appendicular tip should cancer or positive margins be encountered. A functional anastomosis between the distal ileum and transverse colon was successfully completed. Visualization of the peritoneal cavity during the procedure showed no evidence of carcinomatosis, obvious metastatic disease, or malignant ascites.

The patient returned to the office two weeks after the surgery. Pathology from the procedure showed moderately differentiated adenocarcinoma involving the appendix with invasion through the muscularis propria into at least the peri-appendiceal adipose tissue. Carcinoma was present at the mesenteric margin as a solitary tumor deposit, which was felt to be the transected appendiceal tip margin. Perineural and lymphovascular invasion was present. Fifteen lymph nodes were found to be negative for metastatic carcinoma (0/15). Tumor was staged at least a T3 given uncertain involvement of the appendiceal tip and bladder wall. On physical examination, the abdomen was soft, non-tender, non-distended, and had no signs of masses or hernias. The surgical incisions were all clean, dry, and intact without any signs of infection or erythema.

The patient's care was then transferred to an outside hospital for evaluation by Surgical Oncology and discussion of further treatment options. A discussion was held to obtain repeat PET/CT imaging in three months to further evaluate the tumor for possible future en bloc resection with or without hyperthermic intraperitoneal chemotherapy (HIPEC).

## Discussion

Appendiceal adenocarcinoma comprises about 60% of all appendiceal cancer cases but constitutes less than 0.5% of all gastrointestinal tract neoplasms [3]. Adenocarcinoma of the vestigial appendix is divided into the following categories: colonic, mucinous, goblet cell, and signet ring cell types [4]. A preoperative diagnosis is very difficult due to the lack of pathognomonic signs or symptoms and the fact that over 70% of these patients present with clinical symptoms of acute appendicitis. In 2016, an article by Xin Xie reported 1,404 patients with adenocarcinoma of the appendix in the Surveillance, Epidemiology, and End Results Program (SEER) database in China during the period between 2004 and 2013. Of those cases, 48.7% were positive mucinous adenocarcinoma, and 51.3% were non-mucinous colonic-type adenocarcinoma [1].

Acute perforation is one of the complications of appendicitis associated with increased morbidity and mortality. Documented risk factors, especially in the elderly, include male sex, fever ≥ 38°C, diabetes, steroid use, anorexia, and duration of abdominal pain before presentation [5]. Acute perforated appendicitis has been shown to increase in frequency in a linear relationship with the duration of symptoms. The purpose of treating perforations conservatively with antibiotics at the start is to avoid performing an appendectomy in the presence of severe inflammation that obliterates the normal anatomy and creates dense adherence of the surrounding structures. The inflammatory process of appendicitis and the perforation and/or abscess formation is considered in itself to be a predisposing factor for adhesion formation, which complicates future procedures and increases the risk of acute complications including obstructions and strictures. According to Håkanson, a relationship between perforated appendicitis and adhesive small bowel obstruction may be explained by the adhesions formed to nearby structures, and dissection of the inflammatory mass may require more manipulation of the tissue leading to a higher risk of further adhesion formation. Patients with perforation had 9.03 times higher risk of small bowel obstruction (p < 0.001), and patients with the development of an intra-abdominal abscess had 6.98 times higher risk of small bowel obstruction (p = 0.004) [6].

At the time of diagnosis, appendiceal adenocarcinomas can be localized to the appendix or have spread to other regions, including into the peritoneum. Appendiceal adenocarcinoma represents the most common gastrointestinal neoplasm presenting with perforation with some reports indicating a perforation rate of greater than 45%. This leads to the potential for early dissemination in seemingly non-advanced primary lesions. Patients can present with features of acute appendicitis, right lower quadrant mass, urinary frequency, or intussusception. In this case, the appendiceal mass acted in place of a fecalith to occlude the appendix and acted as the catalyst for an inflammatory process in the vestigial organ [7]. Appendiceal cancers that have spread, on the other hand, commonly present with vague abdominal pain and increased abdominal girth. Women may initially present with a presumed pelvic mass, whereas men may present with a hernia that is partially composed of mucin. Due to the poor integrity of the appendiceal wall, cancer cells are able to easily break through its wall and spread into the abdominal cavity and onto the surface of other organs including the omentum, intestines, ovaries, uterus, liver, spleen, peritoneum, and bladder. This spread is known as peritoneal carcinomatosis, and most appendiceal cancers tend to present initially at an advanced stage of this condition [2]. The growth and spread of cancer cells into the peritoneal cavity can lead to decreased appetite, distention, early satiety, nausea, and vomiting. It may also lead to more compact findings such as ascites, pseudomyxoma peritonei (mucinous ascites), or an intestinal blockage. Appendiceal cancer remains a fairly poorly understood gastrointestinal neoplasm. Its cause is not fully understood, and there are no genetic, familial, or environmental factors associated with the disorder. An official diagnosis cannot be made until an excised tumor specimen is examined by a pathologist due to the lack of any unique radiographically identifiable features. In this particular case, diagnosis occurred during right hemicolectomy, but it can occur at the time of appendectomy for acute appendicitis, surgery for an intestinal blockage, or through a diagnostic tumor biopsy for a tumor that was palpable or seen on imaging. The specific subtype of appendiceal tumors and cancers can be distinguished based upon their microscopic cellular composition and specific markers that they stain for. According to recent studies, appendiceal cancers do have a unique genomic profile that is distinct from adenocarcinomas of the colon which may offer future, appendix-specific pathways for treatment [2].

The treatment protocol varies based on the progression of disease and subtype. Appendectomy alone may be sufficient therapy for early-stage tumors in all subtypes except for goblet cell adenocarcinoma. For locally advanced adenocarcinoma, staging is recommended, followed by right hemicolectomy, cytoreductive surgery, and systemic chemotherapy as indicated [2]. Nitecki recommends right hemicolectomy for all non-carcinoid adenocarcinomas due to the risk of overlooked nodal metastases that reach up to 38%. Their studies showed that the survival rate was significantly higher after right hemicolectomy versus appendectomy alone (68% vs. 20%, p < 0.001). Routine oophorectomy in postmenopausal women, regardless of the type of appendicular adenocarcinoma, was also recommended [8]. Chemotherapy for these cancers is often administered in the form of intraoperative HIPEC. The standardized delivery method for colorectal cancer with peritoneal dissemination includes intraoperative delivery of 40 mg in 3 L of perfusate of mitomycin C at the time of cytoreduction using a closed technique. Other options include early postoperative intraperitoneal chemotherapy (EPIC), which is administered in several doses during the first postoperative week, under normothermic conditions via an implanted subcutaneous port [3].

Urological complications are typically inadvertent sequelae that can occur during gynecologic and abdominal surgeries due to the proximity of multiple organs and vessels. If urological complications occur, despite necessary precautions, the assistance of a specialized surgeon should be obtained to effectively repair the complication [9]. In our case, the absence of immediate access to a urological surgeon to assist in the removal of the appendiceal tip from the adhesions attached to the bladder dome and right ureter at our community hospital led to the decision to abstain from attempting to fully exsanguinate the specimen.

## Conclusions

Appendiceal cancer is a rare but plausible gastrointestinal pathology. Due to their difficult diagnostic criteria, histological examination of all appendectomy specimens is strongly recommended to intraoperatively confirm the presence or absence of any tumors that may have predisposed to appendicitis. This case report highlights the atypical presentation of appendiceal adenocarcinoma caused by a complicated appendiceal perforation and its effect on treatment protocols. Critical decision-making by the medical team of their respective hospital's capabilities and limitations is essential to decreasing complication rates and increasing patient survival rates. The recommended treatment for appendiceal adenocarcinoma is right hemicolectomy due to the increased survival rates compared to appendectomy alone. Following a cancer diagnosis, it is recommended to perform surveillance for any synchronous tumors. Treatment options, such as HIPEC with en bloc resection of residual cancer, may be of benefit when the primary tumor is unable to be removed in its entirety with R0 resection.
